# Prevalence of Steroid-Induced Hyperglycemia in King Abdulaziz Specialist Hospital, Taif City, Saudi Arabia

**DOI:** 10.7759/cureus.54430

**Published:** 2024-02-18

**Authors:** Jehan F Sarriyah, Adel S Alghamdi, Norah M Al-Otaibi, Batool B Abdulrahman, Kholoud M Aljaed

**Affiliations:** 1 Internal Medicine, King Abdulaziz Specialist Hospital, Taif, SAU; 2 Endocrinology, King Abdulaziz Specialist Hospital, Taif, SAU

**Keywords:** taif city, diabetes, insulin, hyperglycemia, steroid

## Abstract

Background

Hyperglycemia is a common side effect of high-dose steroid therapy in hospitalized patients.

Objectives

To assess the prevalence of hyperglycemia among hospitalized patients receiving steroid therapy.

Methods

A retrospective study was conducted among 245 patients. The inclusion criteria were patients undergoing steroid therapy and admitted to a single tertiary care hospital due to medical complications or exacerbation of the diseases they were suffering from. Data encompassing patient demographics, admission, discharge dates, comorbidities, medication histories, laboratory results (including blood glucose levels), and documented corticosteroid administrations were meticulously gathered from electronic health records (EHRs). A logistic regression model analysis was done to predict the risk factors of poor glycemic control among hospitalized patients.

Results

The prevalence of hyperglycemia among the patients who were on steroid therapy was 34.2%. About 70.7% of the patients who required insulin at the time of admission required >17 units, and the insulin requirement was significantly higher among patients who received dexamethasone compared to other steroids (p<0.05). Older age (>65 years) was found to be independently associated with poor glycemic control (p<0.05).

Conclusion

The study revealed that almost one-third of patients on steroid therapy had hyperglycemia. Monitoring of patients for hyperglycemia after beginning high-dose steroid therapy should be done.

## Introduction

Steroids are medications that have been used in the management and prevention of a wide range of acute and chronic inflammatory and autoimmune illnesses [1]. Also, steroid therapy, in particular glucocorticoids (GC), has been used in the management of hospitalized patients for various medical conditions [2]. Steroids, such as prednisone or methylprednisolone, are often used to manage inflammatory conditions like asthma exacerbations, chronic obstructive pulmonary disease (COPD) exacerbations, and allergic reactions as these drugs help to reduce inflammation and improve symptoms [3,4].

Steroids are part of immunosuppressive regimens used in patients who have undergone organ transplantation [5]. In cases of septic shock or other types of shock, steroids like hydrocortisone may be administered to support blood pressure and reduce inflammation [6]. Hospitalized patients with severe flare-ups of Crohn's disease or ulcerative colitis may receive steroids to control inflammation and alleviate symptoms [7].

Severe skin disorders, such as severe eczema, pemphigus, or severe allergic dermatitis, may benefit from systemic or topical steroid therapy [8]. During the COVID-19 pandemic, dexamethasone and other corticosteroids have been used to manage severe cases. They help reduce inflammation in the lungs and improve outcomes in some patients [9,10].

Steroids can have several side effects, and one of the significant side effects is hyperglycemia, a condition characterized by elevated blood glucose level [11]. It is a common side effect of high-dose steroid therapy in hospitalized patients [12]. The exact mechanism by which steroids cause hyperglycemia is not fully understood, but it is believed to involve a combination of decreased insulin sensitivity, increased hepatic glucose production, and impaired glucose uptake in peripheral tissues [13].

Steroid hormones are known to have a profound effect on glucose metabolism. They increase hepatic glucose production by promoting gluconeogenesis, glycogenolysis, and lipolysis [14]. Additionally, steroids decrease insulin sensitivity, which leads to impaired glucose uptake in peripheral tissues such as muscle and adipose tissue [15]. These effects are mediated by the glucocorticoid receptor, which is abundantly expressed in the liver, muscle, and adipose tissue [16].

Several studies have provided evidence of the association between high-dose steroid therapy and hyperglycemia in hospitalized patients. For example, a retrospective cohort study conducted by Kim et al. found that the incidence of hyperglycemia was significantly higher in patients receiving high-dose steroids compared to those who did not receive steroids [17]. Another study by Rizza et al. demonstrated that the administration of high-dose steroids resulted in a significant increase in blood glucose levels in healthy volunteers [18]. Moreover, corticosteroids have been shown to impair insulin signaling in peripheral tissues [19].

Insulin resistance in muscle and adipose tissue leads to decreased glucose uptake, and in the liver, it leads to increased glucose production. Corticosteroids have also been shown to impair the insulin secretion capacity of pancreatic beta-cells, which further exacerbates hyperglycemia [20].

Assessing the use of steroid therapy and the incidence of hyperglycemia in hospitalized patients in Saudi Arabia, or any specific region, is an important area of research to understand the local implications and guide clinical practices. This can help healthcare administrators to allocate resources effectively. In addition, this information can guide the provision of diabetes management services and the availability of necessary medications and monitoring equipment [17-19].

This study aimed to assess the impact of steroid therapy on blood glucose level of hospitalized patients due to wide range of chronic diseases in Taif city, Saudi Arabia.

## Materials and methods

Study design and time frame

A retrospective study was done at King Abdulaziz Specialist Hospital (KASH), Taif City, Saudi Arabia in the time from May to July 2023.

Study participants

The inclusion criteria were all adult patients (aged 20 and older) admitted to the study setting and received corticosteroid therapy during their hospital stay.

Data collection

Data were obtained from the electronic health records (EHRs) and hospital databases. A pre-designed checklist was prepared to collect data about patient demographics, admission, discharge dates, comorbidities, medication records, laboratory results (including blood glucose levels), and documented instances of corticosteroid administration. The data collected were carefully entered in standardized proforma by a calibrated investigator. Criteria for identifying patients who received corticosteroids were clearly specified, including searching for specific medication codes or orders within the EHR. Patients with incomplete data were excluded.

Hyperglycemia was defined as fasting blood glucose levels >126 mg/dL or random blood glucose levels >200 mg/dL, consistently applied throughout the study. Ethical approval was obtained from the institutional review board (IRB) to ensure compliance with ethical guidelines, with a focus on protecting patient privacy and confidentiality.

Data analysis and management

Data underwent thorough cleaning and validation to ensure accuracy and completeness. The prevalence of corticosteroid-induced hyperglycemia among the study population was calculated. Subgroup analyses were conducted to explore factors associated with hyperglycemia, including age, gender, corticosteroid dosage, and duration of therapy. Outcomes associated with hyperglycemia, such as length of hospital stay, complications (e.g., infections, diabetic ketoacidosis), and mortality rates, were assessed. Appropriate statistical tests, such as chi-squared tests, correlation test and logistic regression were employed to analyze the data based on the research questions and type of data. The data analysis was done by an independent biostatistician using IBM SPSS version 26 (IBM Corp., Armonk, New York, USA).

## Results

The study included a total of 245 patients who were on steroids and admitted to the hospital due to medical complications. The mean age of patients was 47.1 ± 18.1 years. About 24% (24.9%) belonged to the age group of 36-45 years and 18.2% to >65 years. About 55 (24.4%) were known diabetics and among them, 38 (69.1%) had HbA1c values >6.5%. The anti-diabetic medications used among diabetics (n=55) were as follows: oral agents (41.8%), insulin (40%) and combination therapy (10.9%) (Table 1).

**Table 1:** Baseline characteristics of the patients

Variable		N	%
Age	20-25 years	25	11.1
	26-35 years	44	19.6
	36-45 years	56	24.9
	46-55 years	26	11.6
	56-65 years	33	14.7
	>65 years	41	18.2
Patient is known diabetic	No	170	75.6
	Yes	55	24.4
Last HbA1c values recorded in diabetic patients (n=55)	<= 5.6	2	3.6
	5.7 to 6.4	5	9.1
	6.5 to 8	14	25.5
	8 to 10	13	23.6
	>10	11	20.0
	Not done	10	18.2
Anti-diabetic medication used	Oral agents	23	41.8
	Insulin	22	40.0
	Combination therapy	6	10.9
	Not on diabetic medication	4	7.3

The most common reasons for hospital admission were pulmonary disease (40.9%), and multiple sclerosis (33.8%). The most common type of complication encountered during admission was flare-up of multiple sclerosis (33.3%), bronchial asthma exacerbation (15.6%), COPD exacerbation (9.8%), and systemic lupus erythematosus (SLE) exacerbation (9.8%). About 30 (13.3%) were admitted to the intensive care unit (ICU). The most commonly used steroids were methylprednisolone (61.3%), prednisolone (14.2%), and dexamethasone (13.3%). The mean duration of steroid use was 9.2 ± 67.8 days, and 88% used them for one week or less. The mean random blood sugar (RBS) of the patients was found to be 159.3 ± 92.3, where 24% were found to be diabetic and 10.2% were prediabetic. About 58 (25.8%) received insulin on admission, where 70.7% received >17 units of insulin (Table 2).

**Table 2:** Admission-related characteristics COPD: Chronic obstructive pulmonary disease; SLE: Systemic lupus erythematous; RBS: Random blood sugar.

Variable		N	%
Reason for admission	Anaphylaxis or anaphylactic shock	1	.4
	Hemolytic anemia	1	.4
	Idiopathic thrombocytopenic purpura	9	4.0
	Multiple sclerosis	76	33.8
	Pulmonary disease	92	40.9
	Rheumatologic disease	31	13.8
	Sepsis and septic shock	15	6.7
Type of complication encountered during admissions	Bronchial asthma exacerbation	35	15.6
	Bronchiectasis exacerbation	2	0.9
	Behcet's disease	4	1.8
	COPD exacerbation	22	9.8
	COVID-related complications	5	2.2
	Community-acquired pneumonia	9	4.0
	COVID pneumonia	3	1.3
	Cystic bronchiectasis	2	0.9
	Idiopathic thrombocytopenic purpura	9	4.0
	Interstitial lung disease exacerbation	6	2.7
	Multiple sclerosis flare-up	75	33.3
	Optic neuritis	2	0.9
	Pneumonia	7	3.1
	SLE exacerbations	22	9.8
	Rheumatoid arthritis	3	1.3
	Septic shock	11	4.9
	Lupus nephritis	2	0.9
	Urosepsis	2	0.9
Place of admission	Intensive care unit	30	13.3
	Regular ward	195	86.7
Type of steroid used	Dexamethasone	30	13.3
	Hydrocortisone	20	8.9
	Methylprednisolone	138	61.3
	Prednisolone	32	14.2
	Multiple steroids	5	2.2
Duration of steroid used	Mean duration of steroid use: 9.2 ± 67.8 days		
	<=7 days (1 week)	198	88.0
	7-14 days (2 week)	24	10.7
	15-21 days (3 week)	2	.9
	>2 months	1	.4
Random blood glucose level after administering steroid	Mean RBS (mg/dl)	159.3 ± 92.3	
	Normal (<140 mg‎/dl)	148	65.8
	Prediabetic (140-199 mg‎/dl)	23	10.2
	Diabetic (>200 mg‎/dl)	54	24.0
Patient received insulin during admission	No	167	74.2
	Yes	58	25.8
Insulin requirement for the patient per day (N=58)	Mean units	39.84 ± 29.79	
	5-8 units	5	8.6
	9-12 units	4	6.9
	13-16 units	8	13.8
	>17 units	41	70.7
Sliding Scale Insulin dose	High dose	13	22.4
	Low dose	25	43.1
	Medium dose	20	34.5

It was observed that patients who were on dexamethasone had a significant higher percentage of receiving insulin after admission compared to patients on other steroids (p=0.013) (Table 3).

**Table 3:** Need of insulin at the time of admission based on steroid use

			Steroid					p-value
			Dexamethasone	Hydrocortisone	Methylprednisolone	Prednisolone	Multiple steroids	
Patient received insulin after admission	No	N	16	13	111	22	5	0.013
		%	53.3%	65.0%	80.4%	68.8%	100.0%	
	Yes	N	14	7	27	10	0	
		%	46.7%	35.0%	19.6%	31.3%	0.0%	

When we assessed the duration of steroid use with RBS level, it was observed that diabetics (>200 mg/dl) had a significant higher percentage of patients who used steroids for <=7 days compared to normal or prediabetic patients (p=0.002). Patients who received insulin for >17 units had significant higher percentage of those who were using steroids for <=7 days (p=0.028) (Table 4).

**Table 4:** The RBS level, insulin units administered based on the duration of steroid use RBS: Random blood sugar

Variable			Duration of steroid				
			<=7 days	7-14 days	15-21 days	>2 months	P-value
RBS level (n=245)	Normal (<140 mg‎/dl)	N	134	12	2	0	0.002
		%	67.7%	50.0%	100.0%	0.0%	
	Prediabetic (140-199 mg‎/dl)	N	15	7	0	1	
		%	7.6%	29.2%	0.0%	100.0%	
	Diabetic (>200 mg‎/dl)	N	49	5	0	0	
		%	24.7%	20.8%	0.0%	0.0%	
Insulin Units (n=55)	5-8 units	N	3	1	0	1	0.028
		%	5.8%	20.0%	0.0%	100.0%	
	9-12 units	N	3	1	0	0	
		%	5.8%	20.0%	0.0%	0.0%	
	13-16 units	N	7	1	0	0	
		%	13.5%	20.0%	0.0%	0.0%	
	>17 units	N	39	2	0	0	
		%	75.0%	40.0%	0.0%	0.0%	

**Figure 1:**
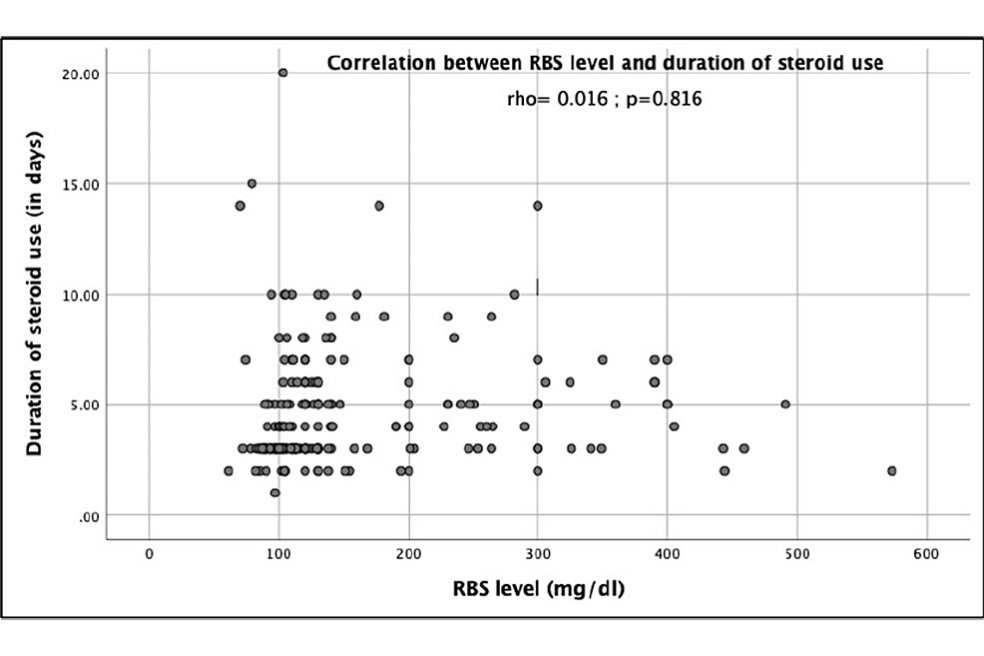
Correlation analysis between RBS level and duration of steroid use RBS: Random blood sugar

A non-significant positive correlation was found between the duration of steroid use and RBS level (rho=0.016, p=0.816) (Figure 1). None of the patients (0%) showed diabetic ketoacidosis (DKA) after corticosteroid therapy.

A multivariate logistic regression model was done to assess the risk factors of poor glycemic control among studied patients (Table 5). It was found that having an age >65 years [OR=3.09 (1.25-5.98), P=0.038] was found to be an independent predictor of poor glycemic control. While receiving insulin of >17 units was an independent predictor of good glycemic control [OR=0.51 (0.12-1.71), P=0.005].

**Table 5:** Risk factors of poor glycemic control in patients on steroids

Variable	Odds ratio	95% Confidence Interval for Exp(B)		P-value
		Lower bound	Upper bound	
Steroid type	0.22	0.00	1.21	0.980
Steroid Dose	0.66	0.02	0.98	0.766
Duration of steroid	0.15	0.00	0.72	0.895
Patient age >65 years	3.09	1.25	5.98	0.038
Type of anti-diabetic medication used	0.00	0.00	0.00	0.999
Patient received insulin during admission	1.32	0.14	2.87	0.987
Insulin units received after admission=>17 units	0.51	0.12	1.71	0.005
Hemolytic anemia	0.08	0.00	2.76	0.987
Thrombocytopenic purpura	5.78	1.06	8.99	0.876
Multiple sclerosis	3.41	0.00	6.72	0.997
Pulmonary disease	0.00	0.00	0.02	0.966
Rheumatologic disease	1.81	0.97	2.85	0.965
Sepsis and septic shock	0.81	0.05	1.23	0.976
In intensive care unit	0.00	0.00	0.02	0.979

## Discussion

Steroid-induced hyperglycemia refers to elevated blood glucose levels (hyperglycemia) that occur as a side effect of corticosteroid medications. Corticosteroids, such as prednisone, dexamethasone, and others, are potent anti-inflammatory medications used to treat various medical conditions, but they can disrupt glucose metabolism in the body [3,4]. Measuring the insulin requirement in patients using steroids is a critical aspect of understanding and managing the impact of corticosteroid therapy on glucose metabolism in a healthy individual. The body regulates blood glucose levels to stay within a narrow range [21]. The findings of our study showed that about 34.2% of the patients who received steroids during admission showed hyperglycemia, and about 70.1% had glucose levels >200 mg‎/dl. The incidence of diabetes mellitus (DM) in individuals without a pre-existing history of hyperglycemia due to steroid use exhibits a range of 34.3% to 56% [22,23]. Various authors have reported a relative risk spanning from 1.36 to 2.31, with a corresponding number needed to harm ranging from 16 to 41 for 1 to 3 years of steroid use [24-25]. A study conducted in the United States in 2017, involving a diverse population, investigated the prevalence of hyperglycemia in both intensive care unit (ICU) and non-ICU patients [26]. The reported prevalence was 32.2% in ICU patients and 32.0% in non-ICU patients. The sensitivity of cells to the effects of insulin is a crucial factor in maintaining glucose homeostasis. Insulin sensitivity refers to how responsive cells are to insulin's actions [27]. In a state of normal insulin sensitivity, only a small amount of insulin is needed to facilitate glucose uptake by cells [28]. Glucose elevation was found to be an independent predictor of poor surgical outcomes, increased hospital stay, and mortality in many observational studies [29-31].

Our findings showed that patients aged >65 had poor glycemic control compared to younger patients. Our results, which are consistent with those of prior case-control studies conducted in an outpatient setting, indicate that the risk of acquiring diabetes exceeds twofold in older patients who are on oral corticosteroids [32-34]. Understanding the heightened risk in older patients is crucial for healthcare providers, as it emphasizes the need for vigilant monitoring and management of blood glucose levels in this population. Additionally, these findings underscore the importance of considering age-specific factors when assessing the impact of corticosteroid therapy on metabolic health. Clinical stratification helps understand patient profiles and determine therapy courses since the severity of the underlying disease varies from patient to patient [35]. Management setting and steroid use are both valid criteria for clinical categorization. Management of hyperglycemia caused by steroid usage requires taking into account a number of variables, including the underlying reason for using steroids, the clinical severity of the disease, the type of steroids utilized, the dose potency being used, and the predicted duration of use of steroids [36]. Nutritional status, organ dysfunction, overall health status, concurrently drugs used, and previous history of diabetes or steroid-induced hyperglycemia are other considerations to take into account [36-38].

Prolonged use of corticosteroids can lead to steroid-induced diabetes, especially in individuals with pre-existing risk factors. Even short-term exposure to steroids can cause hyperglycemia, which may be of concern for patients with and without diabetes. Hyperglycemia, if not managed effectively, can lead to complications such as DKA and hyperosmolar hyperglycemic state (HHS). However, none of the other patients in this study had any such complications. Several factors may contribute to this absence of severe complications. Firstly, the duration of corticosteroid exposure in our study might not have been sufficient to trigger the progression to these advanced stages of hyperglycemia. The risk of DKA and HHS often increases with prolonged exposure to high glucose levels, and the relatively short-term use of corticosteroids in our cohort might not have reached a critical threshold. Secondly, the prompt identification of hyperglycemia during the study period may have enabled timely interventions and management strategies. Proactive monitoring and management of elevated blood glucose levels, especially in a research setting, could have mitigated the risk of progressing to severe complications. Thirdly, the absence of complications might be attributed to the overall health status and vigilance of the study population. It's possible that the patients included in the study had well-controlled underlying conditions or were closely monitored by healthcare professionals, reducing the likelihood of complications. Steroids can lead to insulin resistance through various mechanisms, including altering glucose transport into cells and increasing glucose production by the liver [19,39]. As a result of insulin resistance induced by steroids, more insulin is required to achieve the same level of glucose control that would be maintained in the absence of steroids.

To measure the insulin requirement in patients using steroids, healthcare providers often assess how much exogenous (external) insulin is needed to manage blood glucose levels within a target range [40]. This measurement involves monitoring blood glucose levels regularly and adjusting insulin doses as necessary to maintain glycemic control. Patients receiving corticosteroid therapy may require higher doses of insulin to counteract the insulin resistance caused by the steroids [35]. The insulin requirement is a quantitative measure of this increased need. Quantifying the insulin requirement is essential for tailoring insulin therapy to each patient's needs while minimizing the risk of hyperglycemia or hypoglycemia [41]. It helps healthcare providers make informed decisions about the type, dose, and timing of insulin administration during steroid therapy.

Limitations

The findings relied on existing medical records, which, at times, contained inaccuracies or missing information and might have compromised the precision and comprehensiveness of the data. Despite efforts to control for confounding variables, the retrospective nature of the study made it challenging to account for all potential factors influencing hyperglycemia. Variations in underlying medical conditions, concurrent medications, and lifestyle factors may not have been fully addressed. The study may not have fully determined whether diabetes developed directly as a result of steroid treatment or independently. Some cases of steroid-induced hyperglycemia or diabetes could have gone unnoticed or been misclassified as other types of diabetes, potentially leading to an underestimation of prevalence. Findings from the retrospective study, conducted at a single healthcare institution, might have limited generalizability to a broader population. Variations in patient demographics, clinical practices, and patterns of steroid use may not have been fully considered. The retrospective study was limited in its ability to capture longer-term outcomes or assess the enduring impact of steroids on diabetes beyond the hospitalization period. Being a single center study could hinder the generalization of the study results. Future multicenter longitudinal studies that include larger samples and consider Hemoglobin A1C (HbA1c) level is recommended.

## Conclusions

The findings of the study showed that more than one-third (34.1%) of the patients who received steroid developed hyperglycemia, and among those who required insulin, about 70.7% of patients had an insulin requirement of more than >17 units. Patients who were on dexamethasone received insulin after admission more than others. Older age (>65 years) was found to be independently associated with poor glycemic control and higher doses of insulin therapy. Insulin of <17 units was independently associated with good glycemic control.

A thorough understanding of the mechanisms underlying steroid hyperglycemia is required, as this will enable early detection and effective treatment of affected patients. Patients should be monitored for hyperglycemia after beginning high-dose steroid therapy and treated as needed for effective glycemic control while hospitalized.
